# Medical Society of the County of Chautauqua

**Published:** 1900-01

**Authors:** C. E. Ellis

**Affiliations:** Sherman, N. Y.


### MEDICAL SOCIETY OF THE COUNTY OF CHAUTAUQUA.

REPORTED BY C. E. ELLIS, M. D., Secretary, Sherman, N. Y.

THE semi-annual meeting of the Medical Society of the County of Chautauqua was held at the Hotel Sherman, Jamestown, N. Y., December 12, 1899.

The meeting was called to order by the President, Dr. V. M. GRISWOLD, of Fredonia, at 11 o'clock a. m., when the regular routine of business was transacted in the following order: Reading the minutes of last meeting; admission of new members; unfinished business; reports of committees; miscellaneous business.

The afternoon session convened at 2 o'clock for the consideration of the scientific part of the program: Address of the vice-president—Carcinoma of the stomach and liver—E. A. Scofield, Bemus Point. General discussion. An unusual line of treatment in a case of tetanus, L. C. Green, Panama. Malaria and typhoid fever in pregnancy, William Casper Duke, Cassadaga; Insanity, A. J. Bennett, Busti. Pleurisy, Edgar Rood, Westfield. Report and presenting of interesting cases to the Society. (Each member is expected to report at least one case.) Urea and uric acid from a physiological, pathological and clinical standpoint, Jason Parker, Jamestown.

EVENING SESSION.

Orthopedic appliances, Wm. M. Bemus, Jamestown. Some things I noticed, N. G. Richmond, Fredonia.

The absence of Dr. E. A. Scofield, the vice-president, on account of a serious illness, cast a gloom over the meeting, and his address was therefore omitted.

Dr. F. E. Lilly's death was reported and suitable resolutions were ordered placed upon the minutes, and sent to the family.

In the evening Dr. Wm. M. Bemus read a paper from his personal experience with an orthopedic appliance, he having had to wear one most of his time since he returned from the army. He has secured Mr. E. M. Sheldon to manufacture the one that seems best adapted to his condition.

Dr. N. G. Richmond spoke briefly of a trip abroad he took last summer. He had the attention of the meeting and all were well pleased and instructed by his verbal pictures.

The Jamestown Medical Society entertained the society at a banquet after the evening session. It was served at the New Sherman Hotel, and twenty-four of the members of the societies were present. It was a most enjoyable occasion and one to be long remembered. Those present during the day and evening were Drs. V. M. Griswold, N. G. Richmond, Fredonia; W. C. Duke, Cassadaga; G. F. Smith, Sinclairville; T. D. Strong, Westfield; W. J. French, Hamlet; C. W. Southworth, Forestville; E. S. Rich, Kennedy; W. O. Smith, Falconer; J. R. Smith, Connewango; L. C. Green, Panama; A. J. Bennett, Busti; G. D. Marsh, C. A. Ellis,

Sherman; W. M. Bemus, M. N. Bemus, Jane Greeley, J. J. Mahoney, J. W. Morris, A. B. Rice, L. Hazeltine, H. A. Eastman, A. A. Becker, W. D. Wellman, W. W. Hotchkiss, J. Parker, Jamestown; H. F. Hunt, Dewittville.

